# *Pseudomonas aeruginosa* in Dairy Goats: Genotypic and Phenotypic Comparison of Intramammary and Environmental Isolates

**DOI:** 10.1371/journal.pone.0142973

**Published:** 2015-11-25

**Authors:** Licia Scaccabarozzi, Livia Leoni, Annalisa Ballarini, Antonio Barberio, Clara Locatelli, Antonio Casula, Valerio Bronzo, Giuliano Pisoni, Olivier Jousson, Stefano Morandi, Luca Rapetti, Aurora García-Fernández, Paolo Moroni

**Affiliations:** 1 Università degli Studi di Milano, Dipartimento di Scienze Veterinarie per la Salute, la Produzione Animale e la Sicurezza Alimentare, Milano, Italy; 2 Dipartimento di Scienze, Università degli Studi di Roma “Roma Tre”, Rome, Italy; 3 Centre for Integrative Biology (CIBIO), University of Trento, Trento, Italy; 4 Istituto Zooprofilattico Sperimentale delle Venezie, Sez. Terr. Vicenza, Vicenza, Italy; 5 Institute of Sciences of Food Production, Italian National Research Council, Milan, Italy; 6 Università degli Studi di Milano, Dipartimento di Scienze Agrarie e Ambientali—Produzione, Territorio, Agroenergia, Milan, Italy; 7 Dipartimento Malattie Infettive, Parassitarie e Immunologiche Istituto superiore di Sanità, Viale Regina Elena 299, 00161, Rome, Italy; 8 Cornell University, Animal Health Diagnostic Center, Quality Milk Production Services, Ithaca, New York, United States of America; University of Malaya, MALAYSIA

## Abstract

Following the identification of a case of severe clinical mastitis in a Saanen dairy goat (goat A), an average of 26 lactating goats in the herd was monitored over a period of 11 months. Milk microbiological analysis revealed the presence of *Pseudomonas aeruginosa* in 7 of the goats. Among these 7 does, only goat A showed clinical signs of mastitis. The 7 *P*. *aeruginosa* isolates from the goat milk and 26 *P*. *aeruginosa* isolates from environmental samples were clustered by RAPD-PCR and PFGE analyses in 3 genotypes (G1, G2, G3) and 4 clusters (A, B, C, D), respectively. PFGE clusters A and B correlated with the G1 genotype and included the 7 milk isolates. Although it was not possible to identify the infection source, these results strongly suggest a spreading of the infection from goat A. Clusters C and D overlapped with genotypes G2 and G3, respectively, and included only environmental isolates. The outcome of the antimicrobial susceptibility test performed on the isolates revealed 2 main patterns of multiple resistance to beta-lactam antibiotics and macrolides. Virulence related phenotypes were analyzed, such as swarming and swimming motility, production of biofilm and production of secreted virulence factors. The isolates had distinct phenotypic profiles, corresponding to genotypes G1, G2 and G3. Overall, correlation analysis showed a strong correlation between sampling source, RAPD genotype, PFGE clusters, and phenotypic clusters. The comparison of the levels of virulence related phenotypes did not indicate a higher pathogenic potential in the milk isolates as compared to the environmental isolates.

## Introduction

*Pseudomonas aeruginosa* (family Pseudomonadaceae) is an aerobic, motile, Gram-negative rod, widely present in the environment, e.g. in water and in humid settings [1], but it is also an important opportunistic pathogen for humans, plants and animals. *P*. *aeruginosa* can cause acute and chronic infections in different mammalian hosts and organs, due to the production of a wide arsenal of virulence factors. Virulence factors associated with *P*. *aeruginosa* include flagella, adhesion proteins and extracellular proteins, or secondary metabolites, with proteolytic and/or cytotoxic activity (e.g. exotoxin A, elastase, proteases, pyocyanin, hemolysins) [2]. In humans, chronic infections caused by *P*. *aeruginosa* can persist for months or even years, resisting host defense mechanisms and repeated antibiotic treatments [3].

In animals, *P*. *aeruginosa* is occasionally involved in enzootic or epizootic outbreaks of mastitis in bovine species [4] but also in small ruminants [1, 5]. Small ruminant mastitis is an important issue for veterinarians and animal scientists. Indeed, the number of small ruminant dairies and the consumption of small ruminant milk is increasing worldwide, and the need for producers to provide people with animal proteins in the form of milk and meat will continue to increase [6]. Economic issues derive from production losses, treatment costs, and milk payment policies depending on cellular quality in certain areas [7–9]. In addition, mastitis caused by *P*. *aeruginosa* represents a serious health risk for the human consumer due to possible infections or intoxications through milk, cheese and yogurt [7–9]. *P*. *aeruginosa* infections generally occur in the post-partum period and sometimes during drying-off [1], with either clinical or subclinical intramammary infections (IMI) [5]. Studies performed on *P*. *aeruginosa* strains isolated from clinical and subclinical mastitis in small ruminants and cows analyzed colony morphology, biochemical traits and biofilm production [5].

Recent research regarding phenotypic behavior of small ruminants *P*. *aeruginosa* strains is reported by Wright et al [10]. In this study the authors found that a clone isolated from ovine mastitis showed high expression of genes associated with biofilm and twitching motility [10].

While a high number of studies have been carried out on *P*. *aeruginosa* strains isolated from human infections, few data are available on strains isolated from animal infections. The aim of this retrospective study was to investigate the epidemiology and pathogenicity of *P*. *aeruginosa* isolated from milk and environmental samples in a Saanen dairy goat herd located in Northern Italy, and to analyze their genotypic and phenotypic traits.

## Case Report

An unusual increase in clinical and subclinical IMI caused by *P*. *aeruginosa* in a dairy goat herd owned by the Department of Animal Science, Veterinary College of Milan, was recorded from September 2007 to October 2008; the herd is located in Northern Italy (coordinates 45° 30’ 21.85” N – 9°01’ 17.01” E).

Herd production data were measured monthly by the regional breeding association (Associazione Provinciale Allevatori Milano, Italy) and were referred to the whole period of lactation. These data were made available by Prof. Rapetti, who was responsible of the goats management of the farm. During this period, the herd had an average of 26 Saanen lactating goats, with an average milk production of 58.84 kg per day, meaning an average production per doe from a minimum of 1.14 kg per day to a maximum of 3.38 kg per day; with 3.67% and 3.19% protein and fat content, respectively, and a bulk tank milk somatic cell count of 2.457 x 10^6^ cells/ml. The goats were divided into 4 pens, 3 for lactating animals and 1 as a sick pen. All pens were bedded with straw renewed 1–2 times a week.

In September 2007, 1 goat (goat A) was affected by a severe case of clinical mastitis, characterized by high fever, rapid decrease of milk and atypically associated with diarrheal symptoms. Milk bacteriological analysis showed the presence of *P*. *aeruginosa*. The goat was treated with intramammary tube of nafcillin/benzylpenicillin/streptomycin (Nafpenzal lattazione, Intervet S.r.l., Italy) every 24 h for 2 times and intramuscular treatment with 0.4ml/20kg of tylosin (Tylan 200, Eli Lilly Divisione Elanco, Italy) every 24 h for 3 times and with tolfenamic acid (Tolfedine CS, Azienda Terapeutica, Italy) 1 ml/20kg every 48 h for 2 times. Because these products were not approved by authorities for use in goats in Italy, each drug was administered under Italian regulations (Legislative Decree n. 193 of April 6, 2006) for extra-label drug use (ELDU). After the treatment, a reduction in the clinical symptoms was observed but, at the same time, the monthly Dairy Herd Improvement (DHI) data of this goat revealed a remarkable increase in milk SCC, from an average of 4.150 x 10^5^ cells/ml from April to August 2007, to 4.787 x 10^6^ cells/ml in September 2007. After this single outbreak, all the lactating goats were monitored for 1 year, and milk samples were collected before dry off (November 2007 and October 2008) and after kidding (April 2008). Additional sampling was performed after intramammary antibiotic treatment of the infected animals with cefoperazone in May 2008.

In November 2007, bacteriological analysis of composite milk samples was performed on 22 lactating goats before dry off. Two goats with subclinical IMI (asymptomatic, with high SCC, with no evident macroscopic milk changes and with positive bacteriological analysis) were found positive for *P*. *aeruginosa*, goat A (the goat with previous clinical mastitis) and goat B. The farm manager decided to dry off all the lactating goats with ELDU of 1 tube of nafcillin/benzylpenicillin/streptomycin (Nafpenzal asciutta, Intervet Italy S.r.l) and collect their half udder milk samples immediately after parturition.

In April 2008, post-partum half udder milk sampling performed on 29 lactating goats indicated that 4 other goats with subclinical IMI (goats C, D, E and F) were infected by *P*. *aeruginosa*. Regarding the 2 previously positive goats, only goat A remained infected, still showing high milk SCC (1.316 x 10^6^ cells/ml). Post-partum milk samples from goat B were culture-negative, but with very high milk SCC (3.491 x 10^6^cells/ml) and low milk production (0.7 kg/day), thus the farm manager decided to sell this animal and treat the 5 remaining infected animals. Antimicrobial susceptibility towards 12 antibiotics (i.e. amoxicillin/clavulanic acid, cefalonium, cefapirin, cefoperazone, cefquinome, ceftiofur, danofloxacin, marbofloxacin, nafcillin/benzylpenicillin/streptomycin, neomycin/bacitracin/tetracycline, penethamate and rifaximin) was tested on all isolates, by means of the disk diffusion method. Overall, samples were found sensitive towards only 3 of the tested antibiotics (i.e. cefoperazone, danofloxacin and marbofloxacin). The positive animals were segregated, treated with ELDU of 1 intramammary tube of cefoperazone (Pathozone, Zoetis, Italy) and monitored.

In May 2008, bacteriological analysis confirmed that goats A, C and F remained infected, whereas goats D and E were culture-negative. Since the infected animals showed no clinical signs, only goat C was culled due to its low milk production.

In October 2008, only goat A and another goat (goat G) out of 26 lactating goats were bacteriologically positive, although without clinical signs of infection. The 2 goats were segregated and culled in November 2008 before dry off, in order to avoid further spread of the infection.

Table 1 summarizes *P*. *aeruginosa* detection in goat milk during the study period.

**Table 1:** Time course of the case study. P, positive to *P*. *aeruginosa*; N, negative to *P*. *aeruginosa*; C, culled; NL, not lactating goat.

		Sampling time				
		Lactation	Dry off	Kidding	Lactation	Dry off
Goat	Isolate number	09/2007	11/2007	04/2008	05/2008	10/2008
A	1	**P**	**P**	**P**	**P**	**P**
B	2	N	**P**	N	C	C
C	3	N	N	**P**	**P**	C
D	4	N	N	**P**	N	N
E	5	N	N	**P**	N	N
F	6	N	N	**P**	**P**	N
G	7	NL	NL	NL	NL	**P**

## Results

### Bacteriological analysis

Bacteriological analyses were performed on 142 milk samples (22 composite milk samples and 120 half udder milk samples) collected from September 2007 to October 2008. Of these, 118 (83%) were culture-negative and 24 (17%) were positive. Among the positive cultures, 12 (50%) revealed the presence of *P*. *aeruginosa*, and were derived from 7 goats (27% of the average number of lactating goats). The culture-positive samples showing other bacterial species included 6 samples with Coagulase Negative Staphylococci (CNS) (25%), 2 samples with other Gram-negatives (8.33%), 1 sample with *Streptococcus* spp. (4.17%), 1 sample with *Staphylococcus aureus* (4.17%), and 2 samples with other microorganisms (8.33%, *Micrococcus* spp. and *Bacillus* spp.).

The 12 milk samples positive for *P*. *aeruginosa* had pure cultures (mean 3 x 10^3^ CFU/ml) and a high SCC (mean 5.13 x 10^6^ cells/ml). Statistical analysis performed on the somatic cell score (SCS) revealed a significant difference (*p*<0.05) between culture-negative and *P*. *aeruginosa*-positive samples. Conversely, no statistical difference was observed between culture-negative and other microorganism-positive samples or between *P*. *aeruginosa-*positive and other microorganism-positive samples.

Environmental samples were collected for microbiological analysis in October 2008, after the last 2 infected does were segregated from the herd. This sampling was performed in order to ensure that eliminating all positive animals and maintaining good hygiene practices and biosecurity would reduce the risk of infection from the environment. Environmental samples analysis was also carried out in order to define the sources of the *P*. *aeruginosa* outbreak, and to investigate the interrelationships among milk and environmental isolate genotypic and phenotypic traits.

Out of 41 samples collected from the farm environment, *P*. *aeruginosa* was isolated only from 2 watering trough samples derived from pen number 3 and pen number 4 (sick pen), with 60 CFU/l and 800 CFU/l, respectively. Twenty-six *P*. *aeruginosa* isolates from the 2 watering troughs (disclosing different colony morphotypes) were selected for further analyses, plus 7 *P*. *aeruginosa* milk isolates (1 per infected goat) (Table 2).

**Table 2:** Bacterial isolates characterized in this study.

Isolate number	Sampling date	Sample source	Rapd/PFGE identification	Goat name or trough ♯
1^a^	Sep 2007	Milk	G1/B	Goat A
2	Nov 2007	Milk	G1/A	Goat B
3	Apr 2008	Milk	G1/A	Goat C
4	Apr 2008	Milk	G1/B	Goat D
5	Apr 2008	Milk	G1/B	Goat E
6	May 2008	Milk	G1/B	Goat F
7^a^	Oct 2008	Milk	G1/A	Goat G
8^a^	Oct 2008	Watering trough	G2/C	3
9	Oct 2008	Watering trough	G2/C	3
10^a^	Oct 2008	Watering trough	G3/D	3
11	Oct 2008	Watering trough	G3/D	4^b^
12	Oct 2008	Watering trough	G3/D	4^b^
13	Oct 2008	Watering trough	G3/D	4^b^
14	Oct 2008	Watering trough	G3/D	4^b^
15	Oct 2008	Watering trough	G3/D	4^b^
16	Oct 2008	Watering trough	G3/D	4^b^
17	Oct 2008	Watering trough	G3/D	4^b^
18	Oct 2008	Watering trough	G3/D	4^b^
19	Oct 2008	Watering trough	G3/D	4^b^
20	Oct 2008	Watering trough	G3/D	4^b^
21	Oct 2008	Watering trough	G3/D	4^b^
22	Oct 2008	Watering trough	G3/D	4^b^
23	Oct 2008	Watering trough	G3/D	4^b^
24	Oct 2008	Watering trough	G3/D	4^b^
25	Oct 2008	Watering trough	G3/D	4^b^
26	Oct 2008	Watering trough	G3/D	4^b^
27	Oct 2008	Watering trough	G3/D	4^b^
28	Oct 2008	Watering trough	G3/D	4^b^
29	Oct 2008	Watering trough	G3/D	4^b^
30	Oct 2008	Watering trough	G3/D	4^b^
31	Oct 2008	Watering trough	G3/D	4^b^
32	Oct 2008	Watering trough	G3/D	4^b^
33	Oct 2008	Watering trough	G3/D	4^b^

^a^ sequence for *rpo* gene

^b^ sick pen.

### Isolates genotyping

A positive PCR amplification of the *eta* gene from all 33 isolates, followed by sequencing, confirmed the isolates to be *P*. *aeruginosa*. The isolates selected for *rpo* gene sequencing (Table 2), were submitted to BLAST analysis (http://blast.ncbi.nlm.nih.gov) which confirmed a 100% identity of *P*. *aeruginosa*.

**Fig 1:**
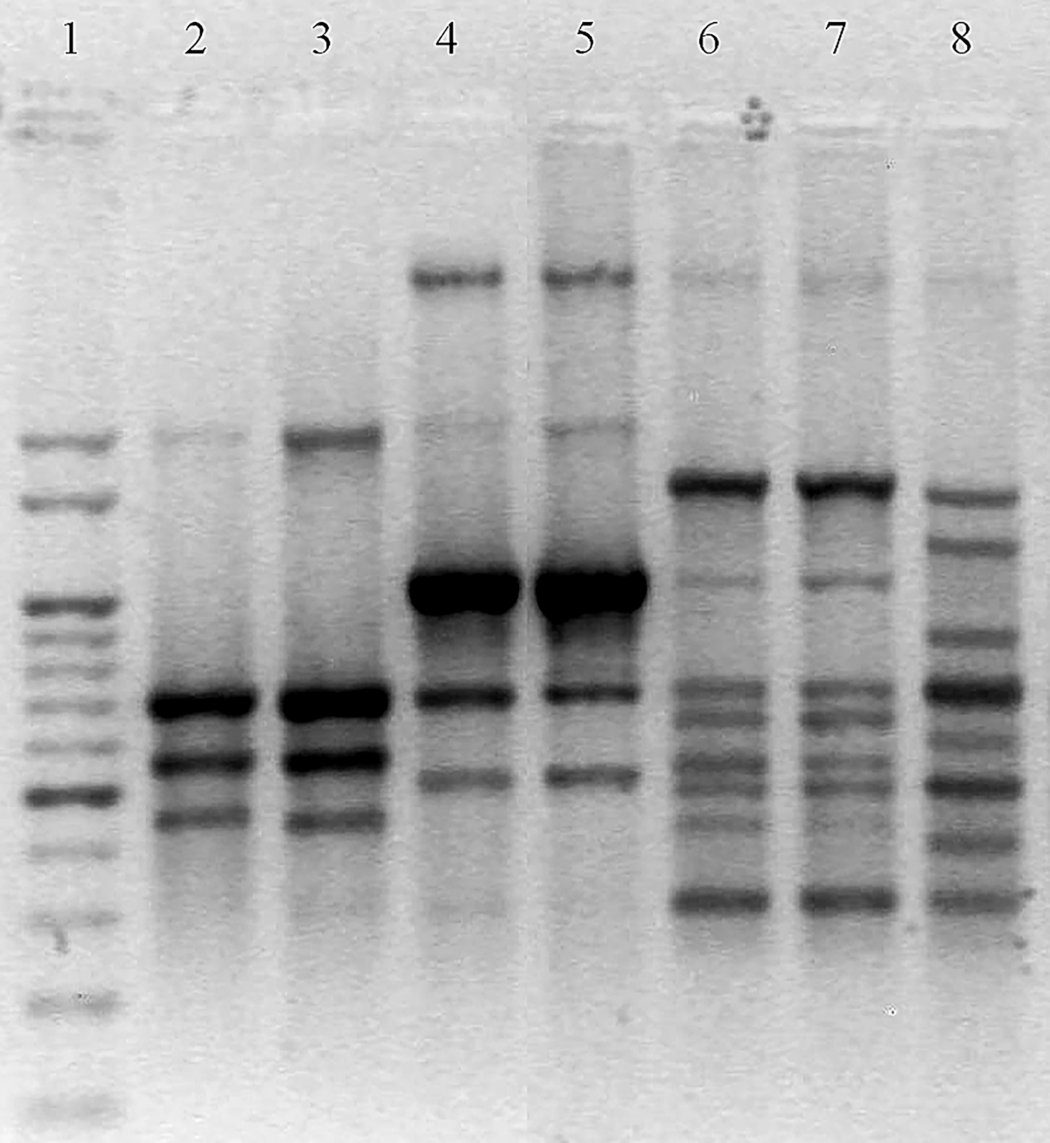
RAPD-PCR banding patterns obtained for the 33 *P*. *aeruginosa* isolates. Lane 1, DNA Ladder; lanes 2–3, milk isolates (isolates 1 and 6, G1 genotype); lanes 4–5, environmental isolates (isolates 8 and 9, G2 genotype); lanes 6–7, environmental isolates (isolates 10 and 24, G3 genotype); lane 8, the human clinical strain *P*. *aeruginosa* PAO1 has been included as a reference. The photograph of the ethidium bromide-stained gel is displayed here as a reverse picture.

The RAPD-PCR assay showed the presence of 3 different DNA-banding patterns (Fig 1). All the milk isolates showed an identical DNA-banding pattern, named G1 (n = 7), while the environmental isolates showed 2 different patterns, G2 (n = 2) and G3 (n = 24).

**Fig 2:**
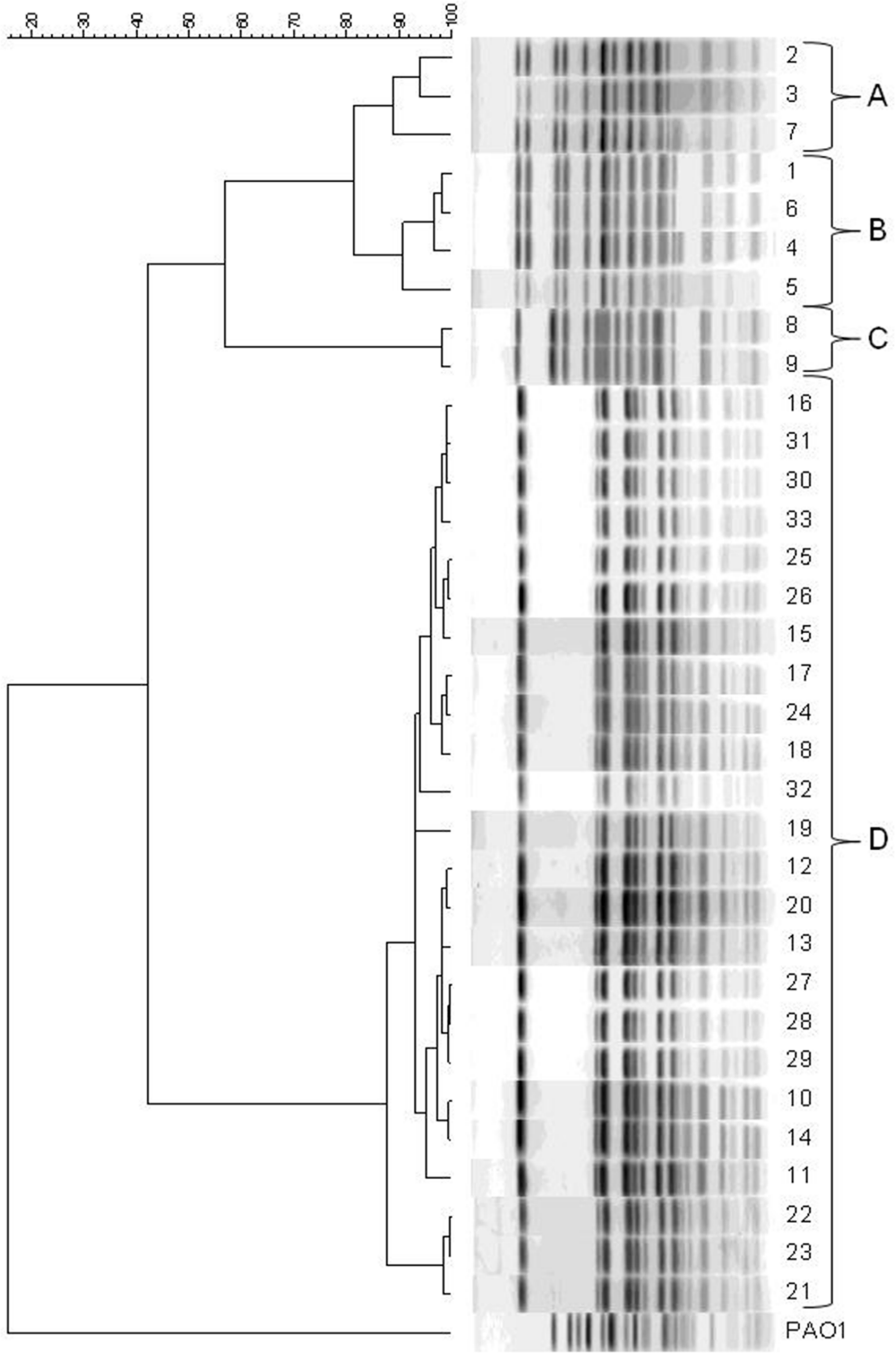
Dendrogram of PFGE-banding patterns of the 33 *P*. *aeruginosa* isolates. Clusters A and B comprise isolates from goat milk, whilist clusters C and D isolates from watering troughs.

In addition to the RAPD-PCR assay, a PFGE assay was performed (Fig 2). By considering a value of ≥85% as the correlation threshold, in accordance with previous studies [11], 4 major clusters were defined. Cluster A (isolates 2, 3 and 7) and B (isolates 1, 4 and 6) shared a high degree of homology (81.45%) and contained milk isolates, while clusters C (isolates 8 and 9) and D (isolates from 10 to 33) contained only environmental isolates. The environmental cluster C showed 56.84% homology with the milk clusters A and B, while the environmental cluster D disclosed a reduced homology (42.15%) with the other 3 clusters.

A high correspondence was found between the RAPD-PCR and PFGE results. Indeed, G1 corresponded to clusters A and B (milk isolates), G2 corresponded to cluster C, and G3 to cluster D.

### Phenotypic characterization of *P*. *aeruginosa* isolates

In order to assess possible correlations between the *P*. *aeruginosa* sampling source, phylogenesis and pathogenic potential, the 33 *P*. *aeruginosa* isolates were tested for the expression of virulence-related phenotypes. The phenotypes included motility (swimming and swarming), hemolytic activity, and the production of biofilm and secreted virulence factors (proteases, pyocyanin, elastase, gelatinase; S1 and S2 Figs). Overall, each isolate showed a unique pattern of production/expression of these virulence-related phenotypes, making it difficult to infer correlations between the pathogenic potential and the phylogenetic cluster and/or with the source of isolation.

### Phenotypic clustering of *P*. *aeruginosa* isolates and correlations within variables tested in this study

**Fig 3:**
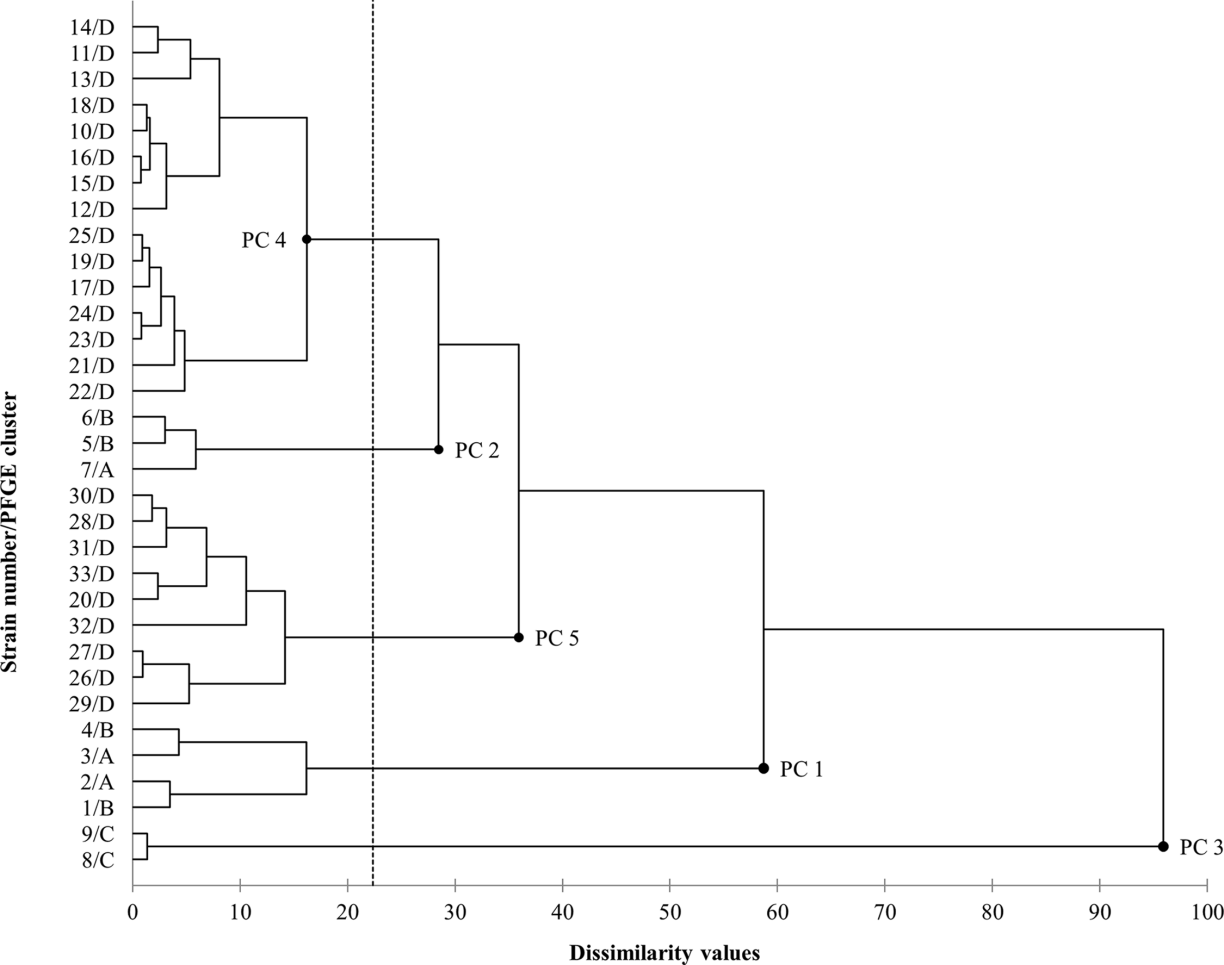
Hierarchical cluster analysis of phenotypic profiles. Dendrogram showing phenotypic clusters (PC 1–5) of the tested *P*. *aeruginosa* isolates. The correlation threshold was automatically set at 22.34% by the software. The vertical axis reports every single isolate number and its PFGE cluster membership.

The phenotypic analysis described above suggested a relevant degree of variance within each genotypic cluster. Hence, clustering aggregation analysis of virulence factor production was performed in order to reveal possible correlations between phenotypes and genotypes. Results showed that the *P*. *aeruginosa* isolates can be divided into 5 major phenotypic clusters (named PC 1, PC 2, PC 3, PC 4 and PC 5, Fig 3), considering a value of 22.34% as the correlation threshold (software automatic truncation). Cluster PC 1 included only milk isolates, *i*.*e*. isolates 2 and 3 (PFGE cluster A) and isolates 1 and 4 (PFGE cluster B), and showed a great phenotypic dissimilarity (58.72%) with the remaining 3 clusters. Cluster PC 2 included the remaining milk isolates, *i*.*e*. isolate 7 (PFGE cluster A) and isolates 5 and 6 (PFGE cluster B), and showed 28.48% of phenotypic dissimilarity with cluster PC 4, the latter including only environmental isolates 10–19 and 21–25 (all belonging to PFGE cluster D). Cluster PC 3 included the environmental isolates 8 and 9 (PFGE cluster C), and showed a high degree of phenotypic dissimilarity (95.92%) with respect to the other clusters. Cluster PC 5 included the environmental isolates 20 and 26–33 (PFGE cluster D) and showed a considerable phenotypic dissimilarity (39.93%) with clusters PC 2 and PC 4. Correlations within variables are shown in the correlation matrix, reporting a high positive association between phenotypic clusters, sampling source and genotypic clusters (Table 3). These high correlations (r>0.7) suggest that genotypic isolate grouping corresponds to a phenotypic one and that this clustering is also in accordance with the sampling source.

**Table 3:** Pearson’s correlation matrix within different variables tested in the study. Bold values are different from 0 at the significance level of alpha = 0.05.

Variables	Source^a^	RAPD^b^	PFGE^c^	PC^d^	Biofilm	Elastase	Gelatinase	Hemolysins	Protease	Pyocianin	Swarming	Swimming
Source^a^	**1**	**0.881**	**0.946**	**0.897**	**0.425**	0.219	-0.287	**0.670**	0.273	-0.141	-0.290	**0.624**
RAPD^b^	**0.881**	**1**	**0.723**	**0.671**	**0.559**	-0.042	**-0.624**	**0.800**	-0.074	0.047	-0.319	**0.551**
PFGE^c^	**0.946**	**0.723**	**1**	**0.914**	0.342	0.325	-0.062	**0.551**	**0.424**	-0.250	-0.187	**0.573**
PC^d^	**0.897**	**0.671**	**0.914**	**1**	0.333	0.191	-0.094	**0.435**	**0.506**	-0.330	-0.039	**0.680**
Biofilm	**0.425**	**0.559**	0.342	0.333	**1**	-0.044	**-0.346**	**0.400**	-0.063	-0.056	-0.100	0.212
Elastase	0.219	-0.042	0.325	0.191	-0.044	**1**	**0.399**	0.072	0.193	-0.132	0.136	0.057
Gelatinase	-0.287	**-0.624**	-0.062	-0.094	**-0.346**	**0.399**	**1**	**-0.397**	**0.440**	-0.210	0.249	-0.124
Hemolysins	**0.670**	**0.800**	**0.551**	**0.435**	**0.400**	0.072	**-0.397**	**1**	-0.278	0.084	-0.118	**0.515**
Protease	0.273	-0.074	**0.424**	**0.506**	-0.063	0.193	**0.440**	-0.278	**1**	-0.247	-0.052	0.031
Pyocianin	-0.141	0.047	-0.250	-0.330	-0.056	-0.132	-0.210	0.084	-0.247	**1**	-0.117	**-0.374**
Swarming	-0.290	-0.319	-0.187	-0.039	-0.100	0.136	0.249	-0.118	-0.052	-0.117	**1**	0.163
Swimming	**0.624**	**0.551**	**0.573**	**0.680**	0.212	0.057	-0.124	**0.515**	0.031	**-0.374**	0.163	**1**

^a^ sampling source

^b^ RAPD genotype

^c^ PFGE clusters

^d^ phenotypic clusters.

Further correlation analyses were performed in order to identify every possible association between the different variables recorded in the study, to find other statistically significant correlations (*p*<0.05), both positive and negative (Table 3). Variables relating to the sampling source, PFGE genotype and phenotypic behavior have certain virulence factors in common, i.e. hemolysis and swimming are virulence factors that are positively correlated (Table 3). Also other virulence factors are correlated (Table 3), indicating possible relationships that would promote specific phenotypic behavior.

**Fig 4:**
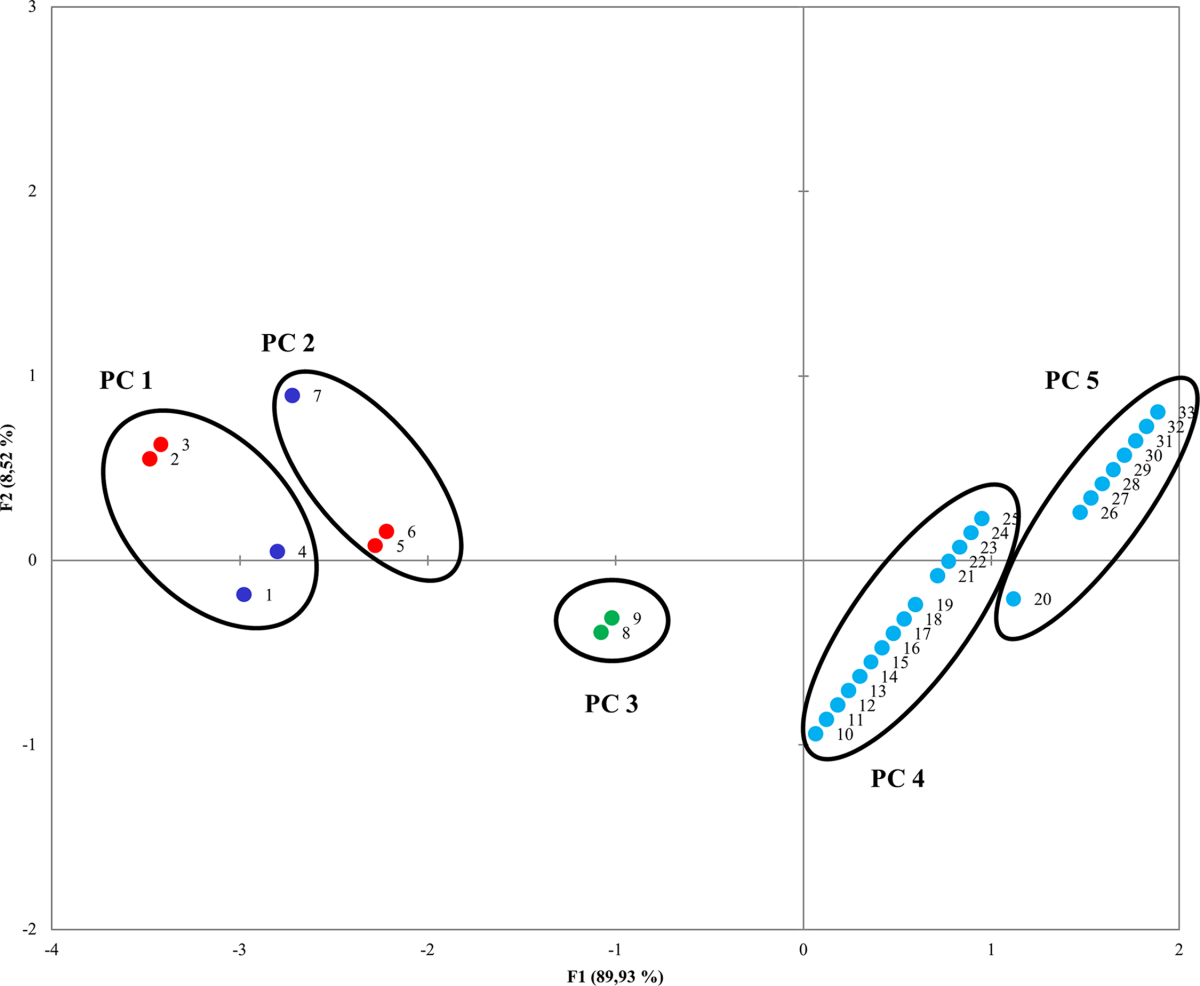
PCA plot showing correlation between phenotypic and PFGE clusters. Phenotypic clusters (PC 1–5) are grouped within circles. PFGE clusters are represented by dots with different colors. Cluster A: red; cluster B: dark blue; cluster C: green; cluster D: light blue.

Principal Component Analysis (PCA) was performed in order to infer the distribution of the 33 *P*. *aeruginosa* isolates in relation to genotypic and phenotypic clusters. Overall, genotypic clustering and phenotypic behavior were significantly correlated. The first two dimensions, plotted on X and Y axes (Fig 4) captured 98.46% of the total variability. The Pearson’s correlation matrix (data not shown) highlighted a consistent association between the analyzed factors (isolates vs PFGE clusters: r = 0.746; isolates vs phenotypic clusters: r = 0.885; PFGE clusters vs phenotypic clusters r = 0.914) (Fig 4).

### Statistical analyses results relating to the production of virulence factors

**Fig 5:**
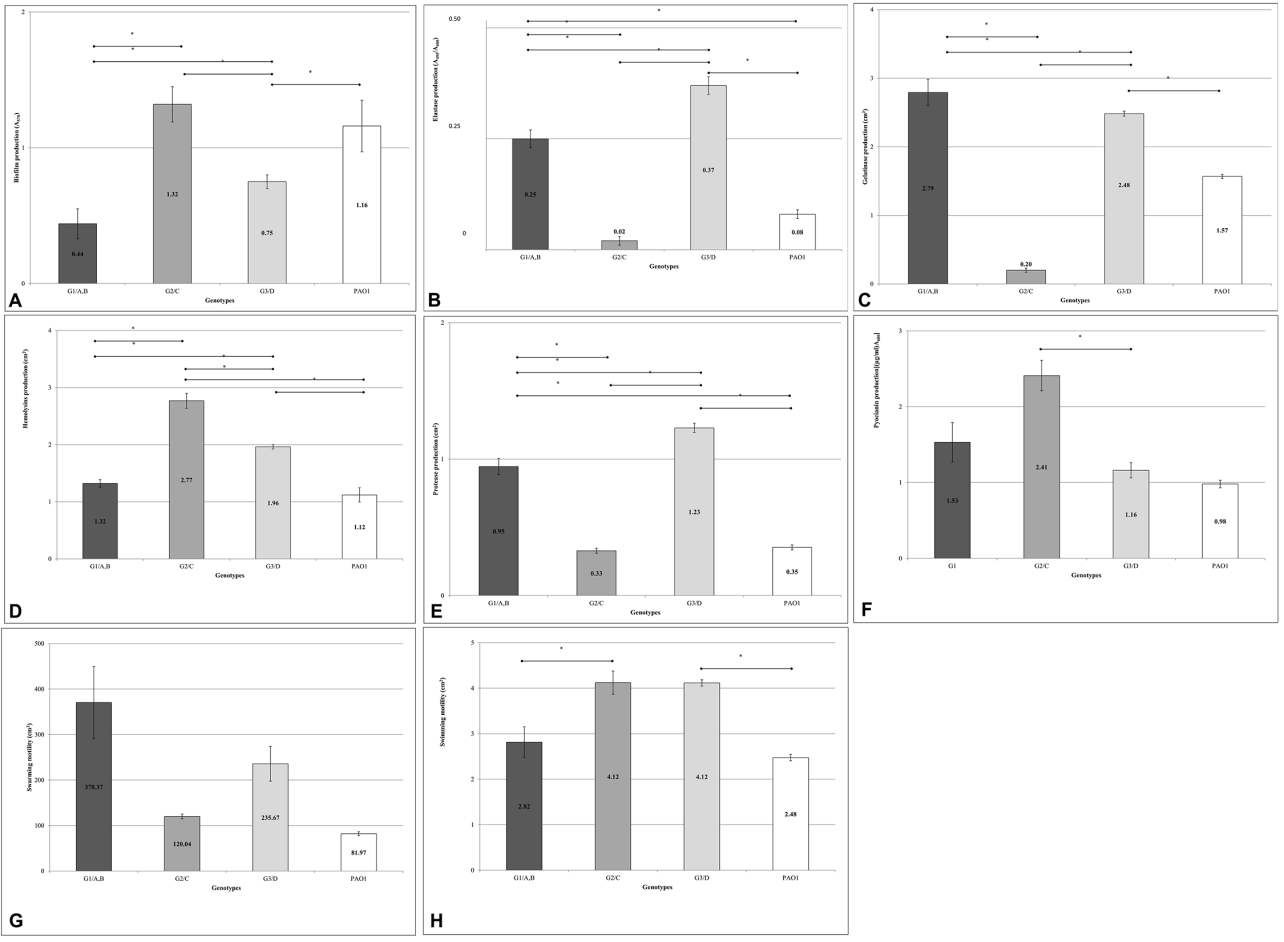
Average virulence factors production by genetic clusters of *P*. *aeruginosa*. Histograms reporting the levels of the indicated virulence-related phenotypes produced, on average, by the *P*. *aeruginosa* strains belonging to the milk isolate cluster (RAPD cluster G1 and PFGE clusters A plus B) and to the environmental clusters (RAPD clusters G2 and G3, corresponding to PFGE clusters B and C, respectively); (A) biofilm production; (B) elastase production; (C) gelatinase production; (D) hemolysins production; (E) protease production; (F) pyocyanin production; (G) swimming motility; (H) swarming motility (H). PAO1 was used as a control strain. The average of the independent experiments is reported with standard errors. Horizontal bars with asterisk indicate *p* < 0.05.

Clustering investigations have highlighted correlations that allowed us to study the average production of each virulence factor for each different genetic cluster (Fig 5). A preliminary analysis showed that the average production of each virulence factor was not significantly different in PFGE clusters A and B (data not shown). This finding is consistent with the high genetic similarity of these 2 clusters, which together include exclusively all the milk isolates. Hence, for the sake of simplicity, the average level of each virulence factor was calculated by considering the milk isolates as a unique group, corresponding to RAPD cluster G1 and to PFGE clusters A and B together (Fig 5).

Overall, the comparison of the levels of virulence related phenotypes, produced on average by each cluster, did not indicate a higher overall pathogenic potential in the milk isolates with respect to the other strains (Fig 5). Indeed, only the average gelatinase production was significantly higher in the milk isolate cluster, while the average levels of other virulence related phenotypes (i.e. biofilm production, hemolytic activity and swimming motility) were lower, with respect to both clusters of the environmental isolates (Fig 5).

### Antimicrobial susceptibility testing and carbapenemase confirmation tests

Minimum Inhibitory Concentration (MIC) of the 33 *P*. *aeruginosa* isolates was analyzed in order to better understand their antimicrobial resistance patterns (Table 4). All the isolates displayed resistance to 3 or more classes of antibiotics. The main pattern of antimicrobial multi-resistance found in 22 of the isolates was penicillin G, oxacillin, ampicillin, amoxicillin/clavulanic acid, cephalosporins I, II, and III generation, macrolides, lincosamides, trimethoprim/sulfamethoxazole, tetracyclines, chloramphenicol. The remaining 11 isolates showed an overall similar pattern of antimicrobial multi-resistance but were susceptible or intermediate to doxycycline. The highest level of resistance was found against the most widely used beta-lactam antibiotics approved in Europe for food animals (ampicillin, amoxicillin/clavulanic acid, oxacillin), and to all the cephalosporins tested. Three isolates from milk (goats A, C and G) were found resistant to imipenem and therefore were tested for the carbapenemase production. All the strains were susceptible to imipenem and meropenem (with an imipenem inhibition zone that ranged from 23 to 25mm and from 30 to 36mm for the meropenem) according to the KPC/ MBL and OXA-48 Confirm Kit and to the Combined-disk method with imipenem and cloxacillin. All the combined-disk tests using the carbapenemase/AmpC inhibitors yielded negative results and Carba NP test did not detect the presence of carbapenemase production. In addition the PCRs for the *bla*_VIM_, *bla*_IMP_, *bla*_GES_ and *bla*_OXA-48_ genes were also negative. None of the *P*. *aeruginosa* isolates met the criteria of multidrug-resistant *P*. *aeruginosa*, which is defined as resistance to both imipenem and gentamicin [12], nor displayed resistance to both of the fluoroquinolones tested (enrofloxacin and marbofloxacin). The resistance to ticarcillin was the only difference found by comparing milk and environment isolates. Overall resistance was higher in milk isolates than in environmental isolates (14% vs 4%).

**Table 4:** MIC of the 18 antimicrobial agents tested for the 33 *Pseudomonas aeruginosa* isolates.

Antimicrobials^1^	Number of *Pseudomonas aeruginosa* isolates with their MIC (μg/ml)											
	0.06	0.12	0.25	0.5	1	2	4	8	16	32	64	128
Ampicillin (0.25–16)			0	0	0	0	0	0	*0*	**33**		
Oxacillin+2%NaCl (0.25–4)			0	0	0	0	**0**	33				
Amoxicillin / clavulanic acid^2^ (4–32)							0	0	*0*	**0**	33	
Amikacin (4–32)							29	4	0	*0*	**0**	
Cefoxitin (2–16)						0	0	**0**	0	33		
Cefpodoxime (2–16)						0	*0*	**0**	0	33		
Ticarcillin (8–64)								0	4	*23*	*4*	**2**
Ticarcillin / clavulanic acid constant 2^3^ (8–64)								0	2	*26*	*3*	**2**
Trimethoprim / sulfamethoxazole^2^ (0.5–2)				0	0	0	**33**					
Cefazolin (4–16)						0	*0*	**0**	0	33		
Gentamicin (1–8)					23	7	*2*	**0**	1			
Imipenem (1–8)					1	20	*9*	**1**	2			
Clindamycin (0.5–4)				0	*0*	*0*	**0**	33				
Penicillin (0.06–8)	0	0	0	0	0	0	0	0	**33**			
Doxycycline (2–8)						0	0	*11*	**22**			
Ceftiofur (0.12–4)		0	0	0	0	0	*0*	**33**				
Marbofloxacin (0.12–2)		0	2	28	2	*1*	**0**					
Enrofloxacin (0.12–2)		0	0	3	*29*	*1*	**0**					
Erythromycin (0.5–4)				0	*0*	*0*	*0*	**33**				
Chloramphenicol (4–16)							0	*0*	*0*	**33**		

^1^ The dilution ranges (μg/ml) tested for each antimicrobial are those within brackets. Values situated above this range indicate the number of isolates with MIC values above the highest concentration tested. Intermediate and resistance breakpoints are indicated with italic and bold formatting.

^2^ Concentrations given for amoxicillin and trimethoprim with a ratio respectively of 2:1 and 1:19.

^3^ Concentrations given for ticarcillin, clavulanic acid concentration is 2 μg/mL in all dilutions.

## Discussion

Outbreaks of *P*. *aeruginosa* mastitis in small ruminants [13–15] and cows [16–18] have occasionally been described. The adaptive behavior of *P*. *aeruginosa* is possible thanks to its genetic flexibility which is supported by an extended genome, containing a high number of accessory genes [3]. Genome sequencing of the extensively studied *P*. *aeruginosa* strain PAO1 revealed the presence of a great number of genes involved in metabolic adjustment, transport, and release of organic elements, as well as numerous chemotaxis systems [19, 20]. These features virtually concur to the notable capability of this bacterium to fit into a wide range of ecological niches [20]. During mastitis outbreaks, *P*. *aeruginosa* has been isolated in contaminated wash hoses in milking parlors, in water and spray nozzles and in contaminated antibiotic preparations [21]. This microorganism is often found in fresh water such as lakes and rivers in concentrations of 100 CFU/l to >10,000 CFU/l [22].

In small ruminants, *P*. *aeruginosa* and other opportunistic pathogens are able to cause enzootic or epizootic outbreaks that mostly appear during the post-partum period and sometimes during drying-off [1]. Additional information regarding udder health is provided by the milk SCC, a valuable tool for the prevalence assessment and screening of IMI in cows and ewes. In goats, the correlation between milk SCC and IMI is difficult to assess, and infection-independent factors play an important role in defining milk SCC values [23]. Hence, in goat herds, it is usually difficult to distinguish infected from healthy udder halves based on milk SCC values only [1]. Nevertheless, *P*. *aeruginosa* positive samples revealed a significantly higher milk SCC when compared to the culture-negative samples, while no difference was found between the negative and positive samples for other microorganisms. This result confirms previous studies showing that infections caused by *P*. *aeruginosa* trigger a greater inflammatory response than other pathogens [24].

After the first outbreak, the milk of goat A, showing clinical signs only at the beginning of the case study, remained positive for *P*. *aeruginosa* throughout the entire period of investigation, indicating the establishment of a chronic infection. In addition, the subclinical goats C and F were positive in 2 consecutive samplings. This confirms previous reports showing that the persistence of subclinical IMI, depending on the causative pathogen and the interaction with the host, is generally high since it is poorly eliminated, at least during lactation, with an average shedding duration of 3–4 months [1].

The persistence of IMI during the dry period must also be taken into account, although an overall self-cure rate of 20% to 60% of halves is estimated in goats [23]. The results reported in this study confirm those recorded in literature [1]. As reported in Table 1, the dry period is focused from November 2007 to April 2008; 2 goats (goat A and B) resulted positive to *P*. *aeruginosa* before dry off (November 2007), while only 1 goat (goat A) still resulted positive after kidding (April 2008), implying that the infection was able to persist during the dry period and through to the subsequent lactation without any clinical manifestation, showing a cure rate of 50%.

A previous study by Contreras et al [8] reported that an infection can be considered a true and persistent IMI when the same pathogen is isolated twice or more consecutively from the same half udder. Accordingly, isolates deriving from goats which were positive in only 1 sample (isolates 2, 4, 5 and 7; Table 1) can be categorized as transient; while those from goats that were positive for at least 2 consecutive samples (isolates 1, 3, 6; Table 1) can be defined as persistent. Statistical analyses were carried out to compare virulence factor production within transient and persistent isolates, finding no significant difference (data not shown).

By examining the phenotypic data, the huge amount of variation existing among each one of the analyzed virulence factors was evident. Each isolate revealed a high degree of dissimilarity from the other isolates, even if belonging to the same genotype. *P*. *aeruginosa* phenotypic changeability is documented in literature, from studies on chronic cystic fibrosis isolates [25]. These isolates, including clones deriving from the same sample, revealed great differences in motility, excreted virulence factors and biofilm formation [25]. Despite this high variation, it was possible to define important phenotypic correlations among the isolates. The degree of genotypic homology confirms the phenotypic profiles of the clusters. Indeed, no significant virulence profile difference was observed between clusters A and B. Therefore, given their common sampling source (goat udder), high genotypic homology and phenotypic similarity, the 2 PFGE clusters were considered to contain isolates belonging to a single ecological niche. This subset corresponds to the G1 genotype detected by RAPD-PCR. The same comparison was made for the environmental isolates, revealing how the lower genotypic homology was related to differences in phenotypic traits. Therefore, it was possible to define the isolates analyzed in the study as having 3 distinct phenotypic features, corresponding to G1, G2 and G3 genotypes (Fig 5). In accordance to a previous study comparing *P*. *aeruginosa* isolates from human (clinical) sources and from the environment [26], no correlation was found between the pathogenic potential of milk isolates (both clinical and subclinical) and environmental isolates.

Altogether, the results obtained by PFGE and RAPD-PCR, well correlated with the phenotypic analysis, revealed that *P*. *aeruginosa* milk isolates belonged to the same clonal line, shared distinctive phenotypic traits and were not retrieved from the environment. These results clearly indicate transmission of the infection within the herd. Although it was not possible to determine the environmental origin of the *P*. *aeruginosa* isolate which caused the outbreak, the observation that all goats were infected by 2 closely related clades of *P*. *aeruginosa* suggests that these 2 clades share a common ancestor particularly well adapted to the udder environment. Since the first goat to show clinical symptoms (goat A) remained chronically infected throughout the entire sampling period, it is not unlikely that the outbreak originated from this animal.

In accordance with data from literature, it is tempting to speculate that such spread occurred during the milking routine, promoted by insufficient cleaning procedures such as teat cleaning with a paper towel and without post-milking teat disinfection [21].

Strikingly, the specific antibiotic therapy with cefoperazone (administered in April 2008) was effective in curing only goats D and E, while goats A, C and F remained infected. The lack of response in the antibiotic therapy of *P*. *aeruginosa* infections is not a rare event, being *P*. *aeruginosa* lung infection a paradigmatic example in human cystic fibrosis [27]. The subsequent analysis of the antimicrobial resistance patterns using the MIC methods showed two main patterns of antimicrobial multi-resistance that included beta-lactam antibiotics and macrolides. These multi-resistance patterns are due to an intrinsic resistance mechanism linked to a reduction in the outer membrane permeability of *P*. *aeruginosa* [28]. The level of resistances found are consistent with the literature data in dairy cattle mastitis that reported a high number of strains resistant to ampicillin, ceftiofur, and 1^st^ generation cephalosporins [29, 30], while no data are available for small ruminants mastitis. Three strains of P. *aeruginosa* were found resistant to imipenem with a MIC of 16 μg/ml for two of them, and of 8μg/ml for the third, but the following carbapenemase phenotypic screening and confirmation tests showed a susceptibility to carbapenems and the absence of carbapenemase production. A possible explanation for this disagreement between the MIC and the carbapenemase confirmation test is that, in the absence of carbapenem-hydrolyzing enzymes, the mechanism leading to carbapenem resistance or reduced susceptibility to carbapenems is usually multifactorial in *P*. *aeruginosa*. Several antibiotic efflux systems, together with AmpC hyperproduction and/or porin loss, contribute to multidrug resistance in this species [31, 32].

Additionally, an important feature that emerged on analyzing the correlation matrix (Table 3) is the positive correlation among the sampling source, RAPD-PCR and PFGE clustering, hemolysins production and swimming motility.

In conclusion, this study shows how caprine *P*. *aeruginosa* isolates could be compared to human isolates, being phenotypically diverse, able to cause both transient and chronic infections, with clinical and subclinical manifestations, and triggering a greater inflammatory response with respect to other pathogens. The study demonstrates that *P*. *aeruginosa* is also able to persist during the dry period through the subsequent lactation, demonstrating the difficult clearance of this pathogen from the mammary gland. Multidrug resistance patterns shown by all the isolates tested assists this resistance to cure IMI. Indeed, the highest levels of resistance were found against the most commonly used beta-lactam antibiotics.

Phenotypic assays showed unique but distinguishable patterns of virulence-related phenotypes. In addition, the combined analysis of data, both as discrete values or as clusters, led to the detection of correlations that would allow the definition of certain isolates as better adaptable to the udder environment. Sampling source, genotypic and phenotypic clustering are highly positively correlated. Association between hemolysins production and swimming motility might suggest that some virulence factors would be capable of making an isolate more adaptable to the goat udder.

One relevant aspect, which broadens the field of interest of this study to beyond veterinary microbiology, emerges when it considering that chronic infections of *P*. *aeruginosa*, in contrast to acute infections, have received much less attention, likely due to the lack of suitable animal models and the difficulty of recreating these long-lasting infections *in vitro* [33]. Up to date, animal models used to study *P*. *aeruginosa* pathogenesis have been mainly based on induced infections in rodents, for example through burns, wounds or direct inoculum of high microbial counts in lungs [34].

This study highlights the fact that increasing knowledge on the pathogenic aspects involved in *P*. *aeruginosa* mastitis in small ruminants could be useful to better understand the behavior of *P*. *aeruginosa* in an animal models where the chronic infection can occur naturally.

## Materials and Methods

### Milk and environmental samples

Milk samples were submitted to the Section of Infectious Diseases, Veterinary College of Milan, for bacteriological culture.

The lactating goats were monitored 4 times by the collection of a total of 142 milk samples, made up of 22 composite samples and 120 half udder samples. No Ethics Committee authorization was required since the milk samples are taken by techniques which are unlikely to cause pain, suffering, stress or any lasting harm equivalent to or greater than the introduction of a needle according to good veterinary practice, as indicated by Legislative Decree n. 26 of March 4, 2014, transposition of Directive 2010/63/EU on the protection of animals used for scientific purposes.

Before milking the animals, teat ends were swabbed with chlorhexidine, dried with a disposable paper towel and, after discarding the first 3 streams, milk samples were collected in sterile tubes. Samples were kept at 4°C and immediately transported to the laboratory for microbiological analysis.

A total of 41 environmental samples were collected from the following sites: goat drinking water (n = 4; 4 from the watering troughs, 1 from each of the 4 pens); goat feed (n = 6; 4 from troughs, 1 from haystack, 1 from pelleted feed); goat resting areas (n = 4; 4 from bedding material, 1 from each pen); goat feces from animals with diarrheal symptoms (n = 10; rectal swab samples); straw used for goat bedding (n = 1); mammary skin from animals with diarrheal symptoms (n = 10); milking apparatus (n = 6; 4 swabs from teat cup liners after milking, 2 from washing water before and after milking). Samples were collected in sterile tubes and immediately refrigerated. Grab samples from bedding and feed were collected in sterile sample bags. Samples were stored in a cooler with ice packs and transported to the laboratory, where processing was initiated within a few hours after sample collection.

### Bacteriological analysis

The milk samples (n = 142) were cultured as recommended by National Mastitis Council (NMC) [35]. A 0.01 ml aliquot of each sample was plated onto 5% sheep blood agar plates (each sample from a goat udder half was placed on a ¼ plate). Plates were incubated aerobically at 37°C and examined at 24 h and 48 h. Single isolated colonies were picked up, subcultured and presumptive identification was performed based on colonies appearance, Gram-staining, catalase and coagulase-testing. Hemolytic staphylococci suspected to be *S*. *aureus* but yielding negative results in the coagulase-test were frozen in nutrient broth with 15% glycerol added before further speciation. For each milk sample, the somatic cell count (SCC) was determined by an automated fluorescent microscopic somatic cell counter (Bentley Somacount 150, Bentley Instrument, Chaska, MN, USA). All bacteriological procedures on the environmental samples were performed on *Pseudomonas* Selective Agar (PSA; Microbiol Diagnostics, Cagliari, Italy). *Pseudomonas* spp. cells from swabs from goat rectums, mammary skin and teat cup liners were directly isolated by streaking on PSA plates. *Pseudomonas* spp. cells were isolated from 100 ml water samples by filtration through 0.22 μm pore size sterile cellulose acetate membrane filters (International PBI, Milan, Italy) and growth on PSA plates. Isolation from bedding and feed was made by adding 10 g of each sample to 90 ml of sterile saline and manually mixing the solution for 10 min. The liquid phase was withdrawn by an automatic pipette, serial 10-fold dilutions were made in sterile saline (final dilution, 1:1,000) and 0.1 ml of each dilution sample was plated on the same medium. Multiple isolated colonies were collected from each plate. The API 20E Diagnostic Kit (BioMerieux, Marcy l’Etoile, France) was used to identify biochemically each isolate to the genus or species level.

### Bacterial isolates

*P*. *aeruginosa* PAO1 was used as a control strain. All bacterial isolates characterized in this study are listed in Table 2.

### Genomic DNA extraction

Genomic DNA extraction from *P*. *aeruginosa* isolates was based on a standard phenol-chloroform extraction. Briefly, all bacterial isolates were grown in Luria-Bertani (LB) broth overnight at 37°C and 1 ml of the bacterial suspension was pelleted at 3,000 g for 10 mins. After supernatant removal, 400 μl of TE Buffer 1X (1.2% Triton), 50 μl of 10% (w/v) SDS, and 50 μl of 20 mg/ml Proteinase K (Novagen, Merck Millipore Italy, Milan, Italy) were used to digest cell pellets at 37°C for 1 h. The lysate was extracted twice with 1 ml phenol:chloroform (1:1, v/v). The upper aqueous phase was added to 1/10 v of 5M sodium acetate and 0.6 v of isopropanol, mixed gently and incubated at room temperature for 5 mins, until the DNA precipitated. The DNA was recovered after centrifugation at 12,000 g for 10 mins, the supernatant was discarded and the DNA pellet was washed with 70% ethanol. After a final centrifugation at 7,000 g for 5 mins, the DNA pellet was dried and finally re-suspended in 100 μl elution Tris-EDTA (TE). DNA concentration was calculated by measuring the absorbance at 260 nm; then 200 ng/μl DNA stock solutions were prepared for each sample.

### PCR for detection of eta gene

P. aeruginosa isolates were identified via PCR using exotoxin A-specific primers, ETA1 (5’-gacaacgccctcagcatcaccagc-3’) and ETA2 (5’-cgctggcccattcgctccagcgct-3’) [36]. The PCR assay was performed in a final volume of 25 μl containing 1x PCR buffer, 2 mM MgCl_2_, 240 μM of each nucleotide, 0.24 μM of each primer (Invitrogen, Life Technologies, Monza Brianza, Italy), 1U Taq polymerase (Invitrogen, Life Technologies, Monza Brianza, Italy) and 2 μl of bacterial DNA template. The chosen amplification protocol was 95°C for 4 mins, followed by 35 cycles of 94°C for 45 secs, 54°C for 45 secs, 72°C for 1 min, and a final extension at 72°C for 5 mins. Ten microlitres of the amplification products were analyzed on 2% agarose gel stained with ethidium bromide in order to evaluate the banding patterns at 367 bp.

### Bacterial typing

Representative isolates selected by PFGE analysis (1 isolate per cluster, Table 2), were submitted to BMR Genomics (Biotechnology Center, Padova, Italy) for targeted sequencing, using PCR primers specific to a region of *rpo* gene [37].

### RAPD-PCR analysis

For typing *P*. *aeruginosa* isolates, primer D-10514 (5’-tggtggcctcgagcaagagaacaaag-3’) was used [38]. RAPD-PCR was performed in a final volume of 25 μl containing 1X PCR buffer, 4 mM MgCl_2_, 200 μM of each nucleotide, 0.5 μM primer (Invitrogen, Life Technologies, Monza, Italy), 1U Taq polymerase (Invitrogen, Life Technologies, Monza, Italy) and 2 μl of bacterial DNA template. Parameters for the amplification were 95°C for 3 mins, followed by 45 cycles of 94°C for 60 secs, 38°C for 60 secs, 72°C for 60 secs, and a final extension at 72°C for 5 mins. Ten microliters of the amplification product was analyzed on 2% agarose gel stained with ethidium bromide in order to evaluate the binding patterns.

### PFGE typing

*P*. *aeruginosa* isolates were also typed by pulsed-field gel electrophoresis (PFGE), as previously described by van Mansfeld et al. [39], with the following modifications. Bacterial isolates were grown in LB broth at 37°C overnight. For each bacterial strain, 3.5x10^8^ cells were mixed with an equal volume of 2% low melting agarose. The resulting plugs were incubated for 5 hours in G-positive lysis buffer (6mM Tris pH8, 1M NaCl, 100mM EDTA-Na_2_ pH 8, 0.5% (w/v) Brij58, 0.2% (w/v) sodium-deoxycholate, 0.5% (w/v) lauroyl sarcosine pH 7.5) with the addition of lysozyme (1 mg/ml final concentration). Plugs were further incubated overnight at 55°C in G-negative buffer (500mM EDTA-Na2 pH9.5, 1% (w/v) lauroyl sarcosine) with the addition of proteinase K (500ug/ml final concentration). DNA restriction was performed for a 2mm plug slice with 20U *Spe*I (Invitrogen, Life Technologies, Monza, Italy), incubating overnight at 37°C. Lambda ladder PFGE marker was loaded in the first lane of each run. Electrophoresis was carried out in 1.2% (w/v) agarose gel in 0.5X Tris-borate-EDTA (TBE) at 12°C on a CHEF DRIII PFGE system (BioRad, Hertsfordshire, United Kingdom). PFGE run setting were: initial switching time 5 s; final switching time 45 s; gradient 6Vcm^2^; 120°C angle; run time 21 h. Samples 6, 7, 12, 34 gave a resolved fingerprint only with electrophoresis in TBE0.5x plus 50uM thiourea. Grouping of the PFGE profiles was obtained with the BioNumerics 5.0 software package (Applied Maths, Kortrjik, Belgium) by using the Pearson product moment correlation coefficient and the UPGMA (unweighted pair group method using arithmetic averages) cluster analysis. Isolates with correlation coefficient > 85% were defined as genetically identical [11].

### Phenotypic assays

The crystal violet binding assay was used to investigate the ability of *P*. *aeruginosa* to produce biofilm and was basically performed as described in [40]. Isolates were grown for 24 h in 5 mL of TSB at 37°C. The absorbance at 600 nm (A_600_) of each sample was normalized to 1.65±0.1. Samples were diluted 1:1 in TSB with 0.25% glucose and 200 μl of this solution was incubated in 96-well plates overnight at 37°C without shaking. Media with suspended bacteria was then removed; the plates were carefully washed four times with sterile water and air-dried before 125 μl of 0.99% crystal violet solution (Merk, Darmstadt, Germany) was added for 15 mins at 30°C. After removing the dye solution and washing four times with sterile water, the attached dye was solubilized with 95% ethanol for 10 mins at room temperature. Absorbance at 570 nm of the solubilized sample was determined by a microtiter plate reader (MULTISCAN EX, Labsystems, Helsinki, Finland). The elastolytic activity in the culture supernatant of the isolates was determined by the Elastin Congo Red (ECR; Sigma-Aldrich, Milano, Italy) assay, as previously described [41]. Isolates were grown in LB at 37°C for 24 h. The absorbance at 600 nm (A_600_) of each sample was normalized to 1.65±0.1. The reaction mixture was centrifuged at 3,000 *g* for 10 mins and the absorbance at 495 nm of the supernatant was determined. A negative control tube containing 0.75 ml buffered ECR and 0.25 ml LB broth and a positive control tube containing 1U Elastase (Elastase, Sigma-Aldrich, Milano, Italy) were used. For measuring gelatinase production, isolates were grown in LB at 37°C for 48 h. The A_600_ of each culture was adjusted to 2.00±0.1. Then, 2 μl of each culture was spotted on LB agar plates containing 5% of gelatins and incubated overnight at 37°C. 600 μl of 15% MgCl_2_ and 20% HCl 1N reagent was added pouring it on plates and turning them to distribute it with homogeneity and, after 3 minutes of incubation, gelatinase production was determined by measuring the area of the circular clear zone around the colonies. Hemolysis was measured by the blood agar assay. Isolates were grown in LB at 37°C for 24 h. The A_600_ of each culture was adjusted to 1.65±0.1. Then, 2 μl of each culture was spotted onto 5% sheep blood agar plates and incubated at 37°C for 48 h. Haemolytic activity was determined by measuring the area of the circular clear zone around the colonies. Protease production was determined by the milk plate assay. Isolates were grown in LB at 37°C for 24 h. The A_600_ of each culture was adjusted to 1.65±0.1. Then, 1 μl of each culture was spotted on Litmus Milk (Oxoid, Milano, Italy) agar plates and incubated overnight at 37°C. The alkaline protease production was determined by measuring the area of the circular clear zone around the colonies. Pyocyanin levels were measured in *P*. *aeruginosa* supernatants as previously described [42]. Isolates were grown in LB broth at 37°C for 48 h. The A_600_ of each culture was adjusted to 2.00±0.1. Samples were centrifuged at 9,000*g* for 10 mins and pyocyanin from 5 ml of the supernatant was extracted with 3 ml of chloroform, centrifuging at 3,000g for 5 min. Pyocyanin was then re-extracted into 1 ml of 0.2 N HCl, to give a pink to deep red solution, and centrifuged at 3,000g for 5 mins. The absorbance of this solution at 520 nm was measured. Concentrations, expressed as micrograms of pyocyanin produced per milliliter of culture supernatant, were determined by multiplying the OD at 520nm by 17.072 [43, 44]. Swarming motility assays were conducted as previously described [45]. Isolates were grown for 24 h in LB at 37°C. The A_600_ of each culture was adjusted to 2.10±0.1. Swarming plates contained 25 ml of the following medium: 0.8% nutrient broth, 0.5% agar, 0.5% glucose. 2 μl of each culture was spotted on the swarming plates and incubated for 24 h at 37°C. Determination of the percentage of swarm plate surface coverage was performed as previously described [45]. Swimming motility assays were conducted as previously described [46]. Isolates were grown for 24 h in LB at 37°C. The A_600_ of each culture was adjusted to 1.65±0. Swimming plates (1% tryptone, 0.05% yeast extract, 0.5% NaCl, 0.3% agar) were inoculated with a sterile toothpick and incubated for 14 h at 37°C. Swimming motility was determined by measuring the areas of the circular turbid zone formed by the bacterial cells migrating away from the inoculation spot. Each sample was tested at least in duplicate.

### MIC and carbapenemase confirmation tests

MIC of 18 antimicrobials (Table 4) were determined for all the 33 *P*. *aeruginosa* isolates using broth dilution test, according to the procedure described in CLSI guidelines VET01-A4 [47]. A commercially available microdilution MIC system, the Sensititre Compan1F Plate Format veterinary panel and Sensititre ARIS system (Trek Diagnostics Systems, East Grinstead, UK) was used. This panel includes antimicrobials and respective MIC dilution ranges listed in Table 4. Results were interpreted using CLSI resistance breakpoints according to M100-S24 guideline [48] for amikacin, doxycycline, imipenem, and VET01-S2 guideline [49] for all the other antimicrobials. For ticarcillin, ticarcillin/clavulanic acid, gentamicin, amikacin and imipenem specific resistance breakpoints for *P*. *aeruginosa* were available, while for doxycycline ampicillin, cefpodoxime, trimethoprim/sulfamethoxazole, enrofloxacin and marbofloxacin were used the resistance breakpoints for *Enterobacteriaceae*. For amoxicillin/clavulanic acid the breakpoint for "other organisms" was used; while for chloramphenicol the breakpoint select was for "organisms other than streptococci". For cefazolin the only breakpoint available was used, that is recommended in CLSI VET01-S2 for all the microorganisms tested. For oxacillin and cefoxitin the only cited resistance breakpoints for *Staphylococcus* spp. were used; for the same reason, for clindamycin the resistance breakpoints for *Streptococcus* spp. was used; for penicillin and erythromycin the resistance breakpoints for *Enterococcus* spp. were used. Finally, the resistance breakpoint for cattle mastitis was used for ceftiofur. The Sensititre plate reading was performed manually recording the last concentration of antimicrobial without turbidity or deposit of cells at the bottom of the well. Considering the great concern for public health induced by the presence of *P*. *aeruginosa* strains producing metallo-beta-lactamases (MBLs) that inactivate carbapenems, the strains that displayed resistance to imipenem, and therefore with a suspected carbapenemase production, were tested by using agar tablet/disc diffusion method by the KPC/ MBL and OXA-48 Confirm Kit (ROSCO Diagnostica A/S, Taastrup, Denmark). A combined-disk method using imipenem and cloxacillin (4000μg), as inhibitor of intrinsic cephalosporinase AmpC, was also evaluated to discriminate between carbapenemase-producing and non-producing *P*. *aeruginosa*. [50]. Carba NP test was performed as previously described [51] in order to detect the presence of carbapenemase. The presence of the most frequent carbapenemase genes found in *Pseudomonas* (*bla*_VIM_, *bla*_IMP_) [52–54] and the *bla*_GES_ and *bla*_OXA-48_ genes were tested by Polymerase-chain-reaction (PCR) [55, 56].

### Statistical analyses

SCCs were transformed to SCS, in order to normalize their distribution using the formula log_2_ (SCC/100,000) + 3 [57]. Statistical analyses were computed using the IBM SPSS 21.0 software for Windows (SPSS Inc., Chicago, IL, USA). SCS, production of biofilm, elastase, gelatinase, protease, hemolysins and pyocyanin, swarming and swimming motility were the response variables. The explanatory variable was the strain relation to a genotype. Data distribution was tested with Shapiro-Wilk test, which found crystal violet biofilm, elastase, gelatinase, protease, hemolysisns, pyocyanin, swimming and swarming data not normally distributed, therefore a non-parametric Mann-Whitney test was used to asses significant differences. On the other hand, Shapiro-Wilk test revealed a normal distribution of data regarding SCS, therefore a one-way ANOVA was used. Levene’s test was used to assess the variance homogeneity [58]. This assumption was satisfied, therefore Bonferroni test was performed. The statistical significance threshold chosen was *p*<0.05. Further analyses were computed with the XLSTAT 2014.6.01 statistical software for Excel (Addinsoft, New York, NY, USA) in order to identify clusters of complex trait phenotypes exhibiting different patterns and discern correlations between different strain sources, genotypes and phenotypic behavior. Phenotypic clustering was estimate through Agglomerative Hierarchical Clustering (AHC) calculating the Euclidean distance and using Ward’s agglomeration method [59]. Correlations within variables tested in the study were obtained through Pearson’s correlation matrix with a significant correlation level chosen at *p*<0.05. Data were also analyzed through PCA in order to visualize significant factors’ correlations (*p*<0.05) on a dimensional map. The analyzed factors were represented by isolates, PFGE and phenotypic clusters.

## Supporting Information

S1 Fig Motility, hemolytic activity and biofilm formation by clinical and environmental *P*. *aeruginosa* isolates. Histograms showing swimming motility (A), swarming motility (B), hemolytic activity (C), biofilm production (D) of *P*. *aeruginosa* isolates. The well-characterized human clinical strain PAO1 is reported as a control. *P*. *aeruginosa* isolates are grouped accordingly to the PFGE analysis shown in Fig 2. Cluster A (milk isolates), black bars; cluster B (milk isolates), dark grey bars; cluster C (water isolates), light grey bars; cluster D (water isolates), white bars. The well-characterized human clinical strain PAO1 (vertical stripes bar) has been used as control, C. The average of three independent experiments is reported together with its standard deviations.
(TIFF)

S2 Fig Secreted virulence factors production by clinical and environmental *P*. *aeruginosa* isolates. Histogram reporting the ability to produce the protease (A), pyocyanin (B), elastase (C) and gelatinase (D) of the tested *P*. *aeruginosa* isolates. PAO1 was used as a control strain. The average of the independent experiments is reported with standard deviations. *P*. *aeruginosa* isolates are grouped by PFGE-banding patterns. Cluster A: black bars, cluster B: dark grey bars, cluster C: light grey bars, cluster D: white bars, PAO1: vertical stripes bar, C. The average of three independent experiments is reported together with its standard deviations.
(TIFF)
