# Supplementary figures and images for: Pseudomonas aeruginosa in Dairy Goats: Genotypic and Phenotypic Comparison of Intramammary and Environmental Isolates
Source: PLoS One. 2015 Nov 25;10(11):e0142973. doi: 10.1371/journal.pone.0142973 (PMC4659641; doi:10.1371/journal.pone.0142973)

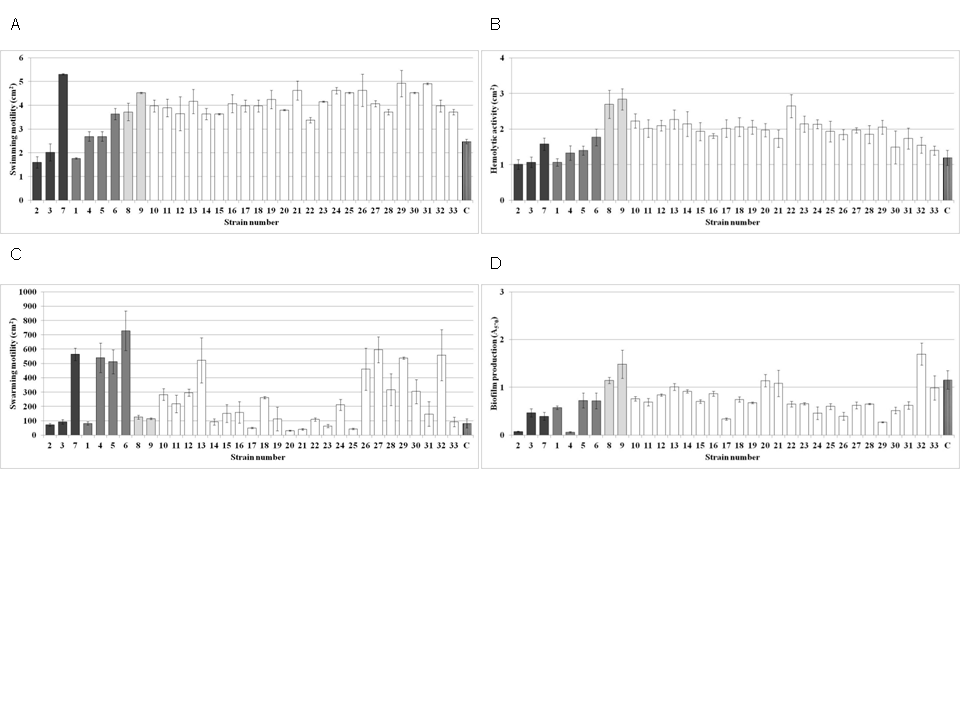

Supplement: S1 Fig — Histograms showing swimming motility (A), swarming motility (B), hemolytic activity (C), biofilm production (D) of P. aeruginosa isolates. The well-characterized human clinical strain PAO1 is reported as a control. P. aeruginosa isolates are grouped accordingly to the PFGE analysis shown in Fig 2. Cluster A (milk isolates), black bars; cluster B (milk isolates), dark grey bars; cluster C (water isolates), light grey bars; cluster D (water isolates), white bars. The well-characterized human clinical strain PAO1 (vertical stripes bar) has been used as control, C. The average of three independent experiments is reported together with its standard deviations. (TIFF) [file pone.0142973.s001.tiff]

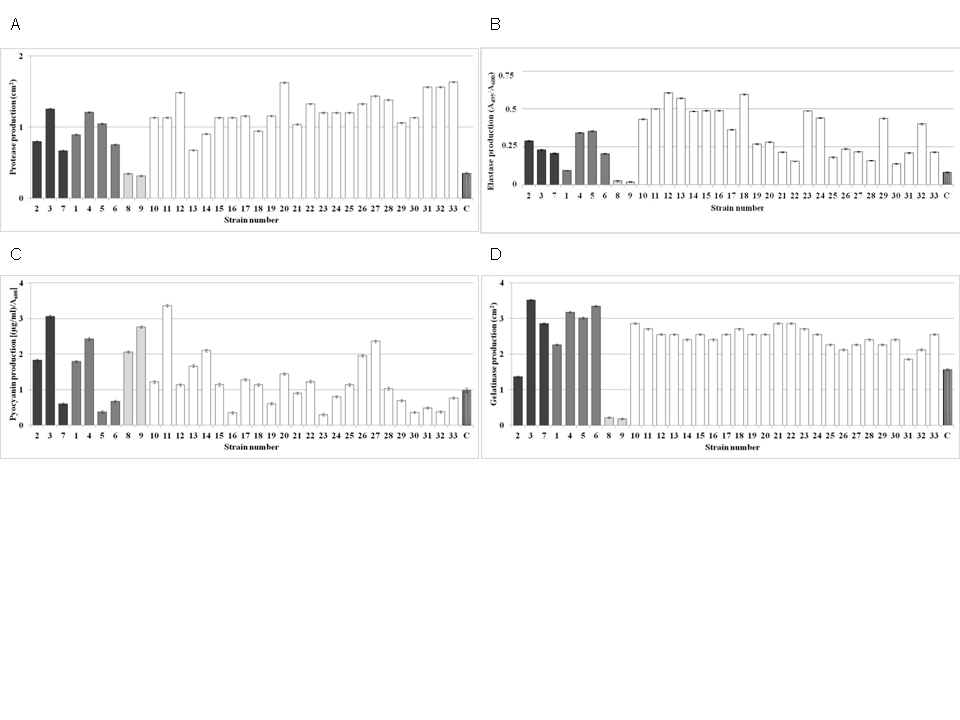

Supplement: S2 Fig — Histogram reporting the ability to produce the protease (A), pyocyanin (B), elastase (C) and gelatinase (D) of the tested P. aeruginosa isolates. PAO1 was used as a control strain. The average of the independent experiments is reported with standard deviations. P. aeruginosa isolates are grouped by PFGE-banding patterns. Cluster A: black bars, cluster B: dark grey bars, cluster C: light grey bars, cluster D: white bars, PAO1: vertical stripes bar, C. The average of three independent experiments is reported together with its standard deviations. (TIFF) [file pone.0142973.s002.tiff]
